# Fecal consumption by adults of altricial birds in relation to the temporal change in nestling gut microbiota

**DOI:** 10.1093/cz/zoaa043

**Published:** 2020-08-13

**Authors:** Li-Fang Gao, Wen Zhang, Hai-Yang Zhang, Zhen-Qin Zhu, Xiao-Dan Zhang, Jian-Chuan Li, Li-Qing Fan, Bo Du

**Affiliations:** 1 School of Life Sciences, Lanzhou University, Lanzhou 730000, China; 2 Tibet Plateau Institute of Biology, Lhasa 850000, China; 3 Institute of Plateau Ecology, Xizang Agriculture and Animal Husbandry College, Linzhi City, Tibet 860000, China

**Keywords:** fecal consumption, giant babax, gut microbiota

Nest sanitation behavior of altricial birds has been selected to reduce the exposure of young to pathogens, parasites, or predators, and thus to maintain the health and safety of nestlings (Guigueno and Sealy 2012). During the process of nest sanitation, caregivers either carry away the fecal sacs of nestlings or directly eat them. Given that adults endure higher risk of being infected by pathogens during fecal consumption (Potti et al. 2007), it remains a puzzle about why adults consume the nestling feces when they can transport them. Several hypotheses have been proposed to explain the fecal consumption behavior of altricial birds, including the nutrition hypothesis (Glück 1988), economic hypothesis (Hurd et al. 1991), and predation hypothesis (Ibánez-Álamo et al. 2013). However, few of them have paid attention to the effect of microorganism on fecal consumption of altricial birds, which are the main component of nestling feces except undigested residue and may contribute to host health (Waite and Taylor 2014). Moreover, it is difficult for current hypotheses to demonstrate why in some species, such as the giant babax *Babax waddelli*, adults consume and even contest the feces of nestlings by adopting cheating strategies (Fan et al. 2017), including pseudo-feeding (without food but mimicking food delivery behavior by touching the gape of begging nestlings), false feeding (delivering plastic debris to the nestlings), and kleptoparasitism (delivering no food but snatching nestling feces after other individuals delivered food). As suggested by the nutrition hypothesis (Glück 1988), if giant babax adults are stressed by innutrition, they should have spent more time on searching for food but not on waiting for nestlings to defecate. After all, the nutritional content in nestling feces is unappealing compared with that in food. Moreover, waiting for a long time at the nest for the nestlings to defecate, but without brooding, will undoubtedly increase the risk of nest exposure to predators, which is contrary to the prediction of economic (Hurd et al. 1991) or predation (Ibánez-Álamo et al. 2013) hypothesis. A recent study in a small-sized mammal suggests that fecal consumption may help herbivores retain gut microbial diversity and function (Bo et al. 2020), to test whether the fecal consumption and contest behaviors of the giant babax is related to nestlings’ gut microbiota, we propose a probiotics-related hypothesis. That is, adults obtain some kinds of probiotics by eating the feces of nestlings so that their gut microbiota might be reshaped during the process of nestling provisioning. This hypothesis seems to explain giant babax’s fecal consumption behavior better than other hypotheses because only very important things are worth contesting. In this study, we first used 16S ribosomal rRNA gene sequencing to identify the major components of microorganisms in the feces of nestlings and adults (Supplementary Table S1). Then, the relative abundances of the top 10 microbial groups (Supplementary Table S2), as well as adult fecal consumption and contest behaviors, were fitted to the nestling age (Supplementary Material). 

During the nestling period, a provisioning adult usually ate the fecal sacs of nestlings. The fecal consumption behavior of dominant females and helpers was significantly negatively correlated with the nestling age (Supplementary Table S3). Dominant females and helpers tended to transport the nestling feces rather than eating them when the nestlings grew to fledge (Figure 1A); whereas the fecal consumption behavior of dominant males did not change with the nestling age, owing to that they consumed the nestling feces until the nestlings were 11 days old, but thereafter they completely transported them (Figure 1A). Regarding the random effects, the intercept of nest identity made a major contribution to the variance of fecal consumption behaviors in all adults (Supplementary Table S3), indicating that most of the variation of fecal consumption behavior was caused by the between-nest rather than the within-nest difference. In some cases, the fecal sacs of nestlings had been eaten by other individuals that delivered no food but waited near the nest for contesting the feces. The fecal contest behavior of helpers was significantly negatively related to the nestling age (Supplementary Table S4), meaning that when the nestlings grew to fledge, helpers did not contest to access the nestling feces (Figure 1B). The fecal contest behavior of dominant females and males did not change significantly with the nestling age (Supplementary Table S4). Regarding the random effects, the intercept of nest identity made a major contribution to the variance of fecal contest behavior of females and males (Supplementary Table S4), indicating that most of the variation in the female or male fecal contest behavior was caused by the between-nest rather than the within-nest difference. In contrast, the slope of nestling age to nest identity made a major contribution to the variance of fecal contest behavior of helpers (Supplementary Table S4), indicating that most of the variation in fecal contest behavior of helpers was caused by the within-nest rather than the between-nest difference. Comparison of the top 10 microbial groups between the feces of nestlings and adults showed that the relative abundances of 4 groups, Proteobacteria, Firmicutes, Acidobacteria, and Chloroflexi, exhibited significant differences between nestlings and adults (Supplementary Table S5). The relative abundance of Firmicutes was significantly higher in nestlings than in adults (*t*_13_* *=* *4.43, *P *=* *0.001), whereas the relative abundances of the other 3 groups were significantly lower in nestlings than in adults (all *P *<* *0.001; Figure 2A). Only the relative abundance of Firmicutes decreased significantly with the nestling age (*F*_1,13_* *=* *60.11, *P *<* *0.001; Figure 2B); the relative abundances of the remaining 9 microbial groups did not change with the nestling age (Figure 2B; all *P *>* *0.05; Supplementary Table S6).

**Figure 1.**
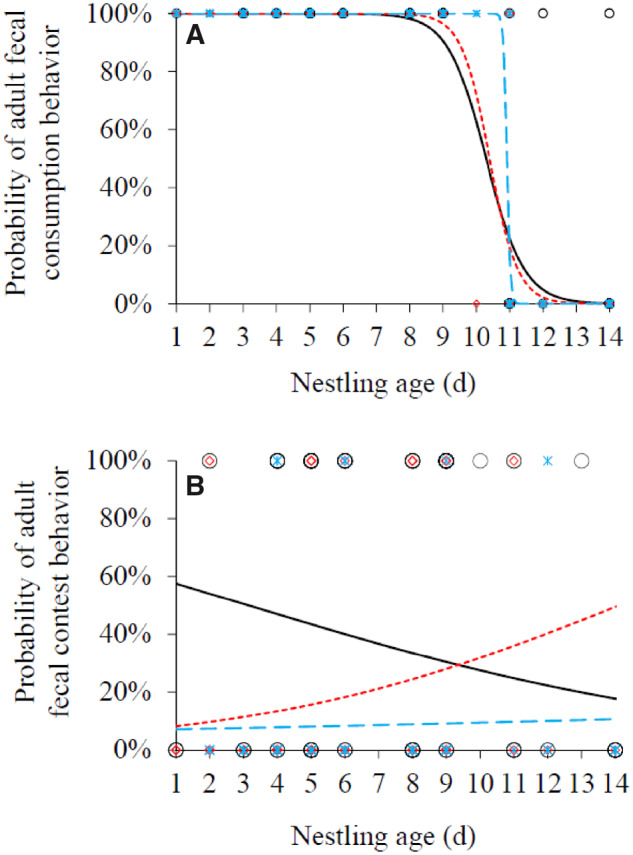
The variation of adult fecal consumption (**A**) and contest (**B**) behavior with the nestling age. Helpers, circles, and solid line; dominant female, diamonds, and dotted line; and dominant male, stars, and dashed line.

**Figure 2.**
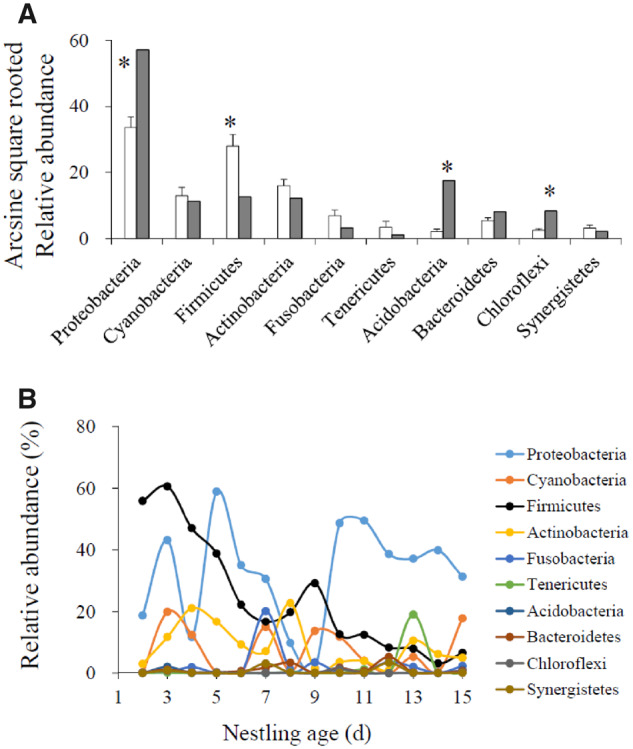
Comparison of the relative abundance of top 10 microbial groups between nestlings and adults (**A**), as well as the variation of their relative abundance with the nestling age (**B**).

Our findings concerning the temporal variation of fecal consumption and contest behaviors of giant babax provide no further evidence for the current hypotheses about fecal consumption in altricial birds. Although there may be residual nutrients or water in the feces of nestlings owing to their lower digestion efficiency (Mcgowan 1995), the amount may be negligible compared with that in the food. Hence, if adults are supposed to obtain nutrients and water as a by-product of nest sanitation, it would be completely unnecessary for the empty-handed visitors to spend time waiting for the nestlings to defecate, let alone adopt cheating strategies to contest for the nestling feces (Fan et al. 2017). Under this condition, to forage and obtain nutrients from food will be undoubtedly a better selection than to spend time on obtaining the nestling feces. If breeders need to perform other parenting behavior, such as brooding, eating nestling feces can save the time required for leaving and returning to the nest (the economic hypothesis, Hurd et al. 1991). From the perspective of dominant breeders in the giant babax, the temporal variation of their fecal consumption behavior conforms to the prediction of this hypothesis. However, it is difficult to understand why helpers that do not brood the nestlings also adjust their fecal consumption behavior with the nestling age. Furthermore, if eating nestling feces is aimed at saving the time and energy required for leaving the nest, it is unnecessary for anyone to contest for the nestling feces when the other group members have prepared to transport them. In addition, the fecal contest behavior of nest-visitors will lengthen the time of staying near the nest and thus increase the likelihood of nest exposure to predators. This behavior cannot be explained by the predation hypothesis (Ibánez-Álamo et al. 2013). In contrast, our probiotics-related hypothesis seems to be more convincing than current hypotheses in explaining the fecal consumption and contest behaviors of giant babax adults. The first evidence comes from the same trends of their fecal consumption or contest behaviors with the nestling age to that of the relative abundance of Firmicutes. Because gut probiotics play a key role in maintaining the stability of the gut microenvironment, especially the Firmicutes in the distal gut that can help the host animals increase their ability to harvest energy from the diet (Waite and Taylor 2014), fecal microbiota transplantation has been suggested as a therapeutic schedule in a variety of chronic intestinal diseases (Leung and Cheng 2019). If giant babax adults can increase the relative abundance of Firmicutes in their gut community by replenishing exogenous Firmicutes from the nestling feces, they may enhance the efficiency of energy harvest during the process of brood provisioning. Another evidence comes from the same trends of adult fecal consumption behavior, as well as fecal contest behavior of helpers, and the decrease in the relative abundance of Firmicutes with the nestling age. The fecal sacs of nestlings are mainly composed of Firmicutes when they are younger, whereas the relative abundance of Firmicutes decreases with the nestling growth. It is thus easier for parental adults to obtain Firmicutes by eating the fecal sacs of younger nestlings than those of older nestlings. Therefore, the probability of adults consuming nestling feces decreased with the growth of nestlings. As Firmicutes are present in the nestling feces but not in the food, it can explain why adults would rather spend more time on waiting for nestlings to defecate than going for forage. Therefore, to obtain exogenous probiotics by fecal consumption or contest may be an adaptive strategy for giant babax to raise their offspring under the harsh conditions in a high-altitude environment.

## Funding

Financial support was provided by the National Natural Sciences Foundation of China (Grant 31572271, 31672299, and 31772465).

## Conflict of Interest

The authors declare no conflicts of interest to any other institutions.

## Supplementary Material

Supplementary material can be found at https://academic.oup.com/cz. 
